# Virulence Mechanisms of *Streptococcus pneumoniae* During Co-Infection

**DOI:** 10.3390/microorganisms14102355

**Published:** 2026-10-14

**Authors:** Nicholas S. Briggs, Stefano Camaione, Jack Goodall, Kevin E. Bruce, Thushan de Silva, Malcolm E. Winkler, David I. Roper

**Affiliations:** 1 School of Life Sciences, University of Warwick, Gibbet Hill Road, Coventry CV4 7AL, UK; 2 Faculty of Infectious and Tropical Diseases, London School of Hygiene & Tropical Medicine, Keppel Street, London WC1E 7HT, UK; 3 Department of Biology, Indiana University, East 3rd Street, Bloomington, IN 47405, USAwinklerm@iu.edu (M.E.W.); 4 School of Medicine and Population Health, The University of Sheffield, Western Bank, Sheffield S10 2TN, UK

**Keywords:** *Streptococcus pneumoniae*, co-infection, molecular mechanisms, influenza, respiratory syncytial virus

## Abstract

*Streptococcus pneumoniae* (pneumococcus) represents a key clinical challenge, being a major global cause of morbidity and mortality. Typically existing as a nasopharyngeal commensal, pneumococcus undergoes significant metabolic changes during its switch to a virulent pathogen, when it can cause severe pneumoniae, bacteriemia and meningitis. This switch is poorly understood but is thought to be influenced by environmental signals arising from co-infections between pneumococcus and other bacteria as well as viruses in the nasopharynx. This review provides a comprehensive overview of the multi-stage progression of pneumococcal invasive disease, from initial colonisation to host barrier migration. We examine environmental triggers that drive the lifestyle switch from commensal to virulent, highlighting the role of other organisms in this stimulation. In particular, we focus on influenza A and respiratory syncytial virus, as well as other bacteria including *Staphylococcus aureus*, *Moraxella catarrhalis* and *Haemophilus influenzae*. Overall, we bring the current understanding of the field into the context of the growing antimicrobial resistance global threat, demonstrating that understanding these complex cross-species interactions is vital for developing clinical interventions against secondary pneumococcal infections.

## 1. Introduction

Microbial infection has been a key clinical challenge since the dawn of civilisation. Traditionally, bacteria and viruses were thought to act in isolation to cause damage to their host. It is now understood that many microorganisms also play important roles in modulating host immunity and the response to infection, either by inducing responses in host cells [1,2,3] or acting directly as pathogens [4,5,6]. However, infections of the respiratory or gastrointestinal tract do not occur in isolation. The human commensal microbiota is present on all mucosal tissues [7], providing a physical barrier to most targets of invading viral or bacterial infections, as well as a competitive environment for growth.

It has been noted for many years that bacterial cross-species communication occurs in biofilm settings [8,9,10]. The relationships between members of a biofilm are generally mediated by either direct protein–protein interactions that drive signalling cascades within the target cell [11] or by one of many secretion system assemblies delivering toxins into the target cell [12,13]. Quorum sensing also plays a crucial part in this interplay through the release, detection and response to small molecule autoinducers [14,15]. Through these mechanisms and others discussed below, bacteria are able to adapt to changing environmental signals, which in turn can change the trajectory of disease progression.

However, the field is now developing a better understanding of the role of interkingdom relationships (bacterial/viral or bacterial/host) that influence the virulence mechanisms of bacterial pathogens. These understandings may drive future development into next-generation antibacterial therapies, though they are currently underrepresented in the literature. This review aims to compile the current understanding of some interkingdom relationships in the context of respiratory infection.

### 1.1. Streptococcus pneumoniae: An Opportunistic Pathogen

One notable commensal species for which this changeable progression is particularly important is the human opportunistic pathogen *Streptococcus pneumoniae* (pneumococcus). This Gram-positive organism represents one of the greatest threats to human health globally, with the WHO regularly including the bacterium on its list of priority pathogens since 2017 [16,17]. This pathogen has also been identified as one of the five species responsible for half of the global deaths associated with bacterial infection [18], causing a range of diseases including pneumonia, otitis media and meningitis, demonstrating its wide range of targets within the host [19]. Despite significant reductions in the incidence of both invasive pneumococcal disease (IPD) and community-acquired pneumonia (CAP), principally attributable to the widespread adoption of pneumococcal conjugate vaccines [20], pneumococcus continues to be a significant cause of morbidity and mortality globally. The most recent Global Burden of Disease found this pathogen to be both the most common etiological cause of lower respiratory tract infection (24.2% [95% CI 23.5–24.9]) and the leading cause of death (25.3% [95% CI 24.5–26.1]) in all ages, with higher proportions of both children under 5 and the elderly [21,22].

These developments are likely influenced in part by the bacterium’s natural competence mechanisms [23]. The ability to uptake environmental DNA creates an efficient model for acquiring antibiotic resistance across the entire local population of pneumococcus, allowing it to outcompete many other species in mixed-population biofilms and cultures [24]. Moreover, *S. pneumoniae* represents the traditional model for capsule formation [25,26], whereby polysaccharide chains are anchored directly to the peptidoglycan layer of the cell wall via the actions of the Wzy pathway [27,28]. Changes in these sugars denote a change in pneumococcal serotype and contribute directly to virulence and disease severity [29,30], owing largely to the role of the capsule as the principal shield between bacteria and host defences [31,32].

### 1.2. Current Clinical Interventions

Treatment of pneumococcal infections has been focused on prevention for several decades, with antibiotics being used following symptom presentation. Vaccination has become a critical intervention in the prevention of pneumococcal disease globally. Many vaccine programmes have moved away from purified capsular polysaccharide-based vaccines like PPV23 [33], replacing them with attenuated diphtheria toxin-conjugated sugars [34]. This formulation, whilst reducing the serotype coverage, generates a higher efficacy vaccine, with some studies highlighting increased longevity of protection in high-risk subjects [35,36]. Whilst this increase in effectiveness is promising compared to PPV vaccines, these formulations suffer from specificity issues given they currently cover a maximum of 21 of the more than 100 known serotypes [37]. Where clinical disease develops, optimal antimicrobial management is nuanced, depending on the severity of illness, the site of infection, the antibiotic resistance profile, and patient-specific factors such as allergies, drug interactions, or immunodeficiencies [38,39]. As a result, vaccination is considered the cornerstone of prevention currently, with antibiotics being used for treatment. It should be noted, however, that in the context of co-infections discussed later, most treatment options are additive, not synergistic.

### 1.3. Antimicrobial Resistance—A Global Threat

The rise of antibiotic resistance in pneumococcus is rapidly eroding the pool of effective treatments. Recent reports have revealed a dramatic rise in multi-drug resistant (MDR) strains as well as a significant emerging threat of strains resistant to an extensive list of antibiotics (XDR) [40,41,42]. This trend is especially alarming given the ecological context of pneumococcal infection. As an opportunistic commensal bacterium, pneumococcus lives within a complex ecological niche where its growth and infection dynamics are shaped by environmental cues from co-resident organisms. As discussed above, pneumococcus is remarkably adept at harvesting DNA from its environment and, as a result, often incorporates genes of evolutionarily related commensal microbiota associated with antibiotic resistance [43]. This horizontal gene transfer in turn amplifies the threat of drug resistance development as well as changes in infectivity profiles.

However, pneumococcus appears to be influenced by multiple signals in addition to gene transfer, which are often generated directly from other pathogens, including specific respiratory viruses (discussed below). These mechanisms are not fully understood but appear to hinge on nutrient supply as well as local inflammation driving changes in pneumococcal gene expression (discussed below). Specifically, local temperature changes appear to trigger upregulation in surface adhesins, which is accompanied by increased detection of nutrients, which in turn upregulates key virulence factors such as pneumolysin, as well as other mechanisms outlined below. This review will provide an overview of *S. pneumoniae* infection dynamics and how these are influenced by other commensals and pathogens during co-infection.

## 2. Pneumococcal Infection Overview

**Figure 1:**
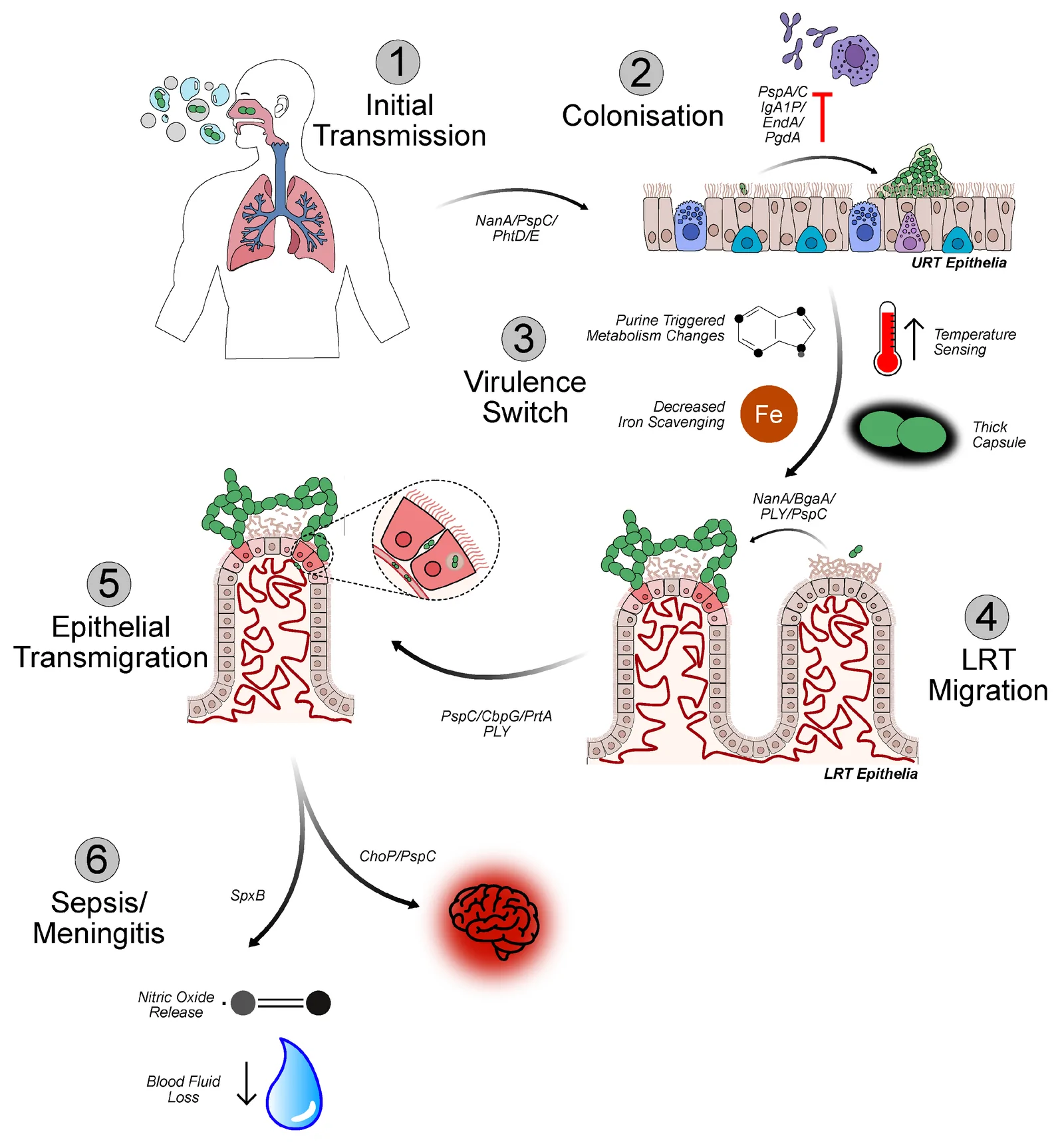
Graphical summary of pneumococcal infection. Inhalation of bacteria-containing aerosolised droplets (**1**) allows pneumococcal colonisation of the URT (**2**). Following ineffective immune clearance, pneumococcus establishes itself as a commensal bacterium before environmental triggers induce a switch to a virulent state (**3**) and disperses from the nasopharyngeal biofilm to the sterile lining of the alveoli in the lower respiratory tract (**4**). Following colonisation and sputum production, pneumococcus migrates across the basal epithelial layer (**5**), enters the bloodstream (**6**), and potentially crosses the blood–brain barrier.

The first exposure to pneumococcus typically occurs within the first months of life [44,45]. This is thought to most commonly occur through respiratory droplets from the mother during close contact with the infant, though rare cases have been documented of infections resulting from colonisation of the vaginal canal during birth [46,47,48], or via ascending intrauterine infections [49]. Cases of perinatal infection have high mortality rates (>50%) as a result of neonatal sepsis [50]. In general, humans carry the bacteria intermittently and asymptomatically within their nasopharynx, serving as the main source due to the lack of other major zoonotic reservoirs (e.g., serotype 3 can be found in horse populations [51]). The following section outlines the current understanding of the stages of pneumococcal infection, which are summarised in Figure 1.

### 2.1. Initial Adherence and Colonisation

Pneumococcus spreads primarily through respiratory droplets, which are transmitted directly from person to person through close contact [52]. This allows pneumococcus to colonise the URT of a new host, initially binding to mucin, likely via neuraminidase-mediated mechanisms, as demonstrated in murine models [53]. This interaction serves both to allow pneumococcus to adhere to mucosal surfaces and release beneficial nutrients. To prevent pneumococcus from becoming trapped in this mucus layer and subjected to host defences (e.g., lysozyme, HPN-1, etc.), anionic capsular polysaccharides induce a charge-based repulsion against mucosal polysaccharides [54]. These effects combine to allow pneumococcus to translocate to the host epithelial surface, where it begins to colonise the apical surface via the actions of various adhesin proteins, including PspC [55,56], PhtD/E [57,58] and NanA [59]. Neuraminidase activity is key to success at this stage, as they bind to and cleave sialic acid from host surface glycoproteins as the first step, releasing sugars as a carbon source for pneumococcal growth [60,61]. It should be noted that many virulence factors mentioned in this work are not exclusively bound to the single function described, as they can be classified as moonlighting proteins, presenting multiple, often overlapping, functions [62].

Adherence alone, however, is not sufficient for persistence. Many proteins involved in adhesion also have roles in immune evasion or modulation, allowing pneumococcus to maintain its population as a biofilm within the nasopharynx [63]; this stage is critical to bacterial survival in the host environment. To maintain this state, several protein families are involved. The major surface protein PspC has been found to bind directly to complement component C3 in vitro, whereas PspA is thought to inhibit deposition of C3 on the pneumococcal cell surface [55,64,65,66]. This in turn leads to a significant decrease in inflammatory responses and opsonophagocytic clearance. Concurrently, increased levels of an IgA protease, IgA1P, occur, which cleaves the protective antibody [67], leading to subversion of protection and decreased opsonophagocytosis [68]. Additionally, the endonuclease EndA is able to degrade neutrophil extracellular traps (NETs), thereby preventing entrapment [69,70]. Interestingly, EndA activity has been shown to be independent of the competence apparatus, and it has been suggested to be secreted into the surrounding environment through post-translational cleavage of its transmembrane (TM) helix [71,72]. Additionally, the metal-dependent PG deacetylase PgdA removes N-acetyl groups required for host lysozyme activity in vivo [73,74].

### 2.2. Switch from Commensal to Pathogen

These protective measures allow pneumococcus to establish a “beachhead” within the URT, evading host immunity and persisting as a commensal bacterium. In many instances, pneumococci will either remain in this commensal state or be cleared by the immune system. However, certain instances will lead to a transition from commensal to invasive pathogen through triggers that are not fully understood. Several key events have been implemented in this switch that appear to be linked to co-infections, particularly viral. Firstly, the result of the primary infection may trigger a fever response, raising the URT from around 32 °C to closer to 37 °C. Should this happen, an RNA thermosensor system built into the 5′ UTR of *ciaR* (a component of the *ciaRH* system) is stimulated [75,76] to control around 17 downstream genes [77,78]. This stimulation unfolds a hairpin regulator, allowing the RBS to become fully accessible [76]. This upregulation leads to increased synthesis of surface teichoic acids. Through molecular mimicry of host PAFR, these phosphocholine residues stimulate bacterial invasion by increasing host adhesion to PAF receptors in murine models [76]. Following attachment, pneumococcus likely can respond to exogenous, host-derived ATP and noradrenaline released during viral infection [79,80,81], which could provide a trigger for dispersion of virulent pneumococci from the biofilm.

Increased ATP has been reported to influence the pneumococcal transcriptome, including altering influx of purine compounds through changes in membrane potential, which in turn triggers downregulation of the competency regulon [82,83,84]. This, coupled with the increase in sugar and lactate metabolism, provides the energy requirement for pneumococcus to dismantle its biofilms and disperse [84]. Whilst this response to ATP is more associated with changes to metabolism, noradrenaline is detected by a specific two-component system, TSC09 [85], as well as in part by PspA and PspC [86]. Combining a sensor histidine kinase (HK09) and a response regulator (RR09), this system increases migration into the lower respiratory tract (LRT) via two distinct systems [86,87]. Transcriptional regulators alter capsule production to produce a much thinner layer to expose more surface adhesins, whilst glycosidases (NanA/B) and an iron transporter (PiuA) are upregulated [85], which in turn could increase growth rate and dispersion. The thicker capsule here is particularly important in preventing phagocytosis by macrophages.

Iron transport also plays a key role in pneumococcal virulence. In general, the immune system attempts to “starve” bacteria by sequestering free iron, thereby shutting down metabolic pathways and DNA replication in vivo [88]. Pneumococcus has several systems to compensate for this (e.g., the ABC transporter Pit [89]), including scavenging surface proteins like PspA [90], as well as specific two-component systems (TCS08). Interestingly, iron scavenging may be linked to hydrogen peroxide production, which pneumococcus makes as a byproduct of its own metabolism [91,92]. Whilst this has obvious implications in local tissue damage during infection, hydrogen peroxide can also readily interact with iron, both free and haem-bound [93]. This is thought to increase the levels of available iron around pneumococcal cells for import whilst also inhibiting competing bacteria in the local niche [94]. Orphan response regulators (RitR) regulate a variety of iron import machineries, including repressing PiuAB in high hydrogen peroxide environments [95,96]. During colonisation, the SpxB pathway is activated and has been shown to be essential for biofilm production in vivo [97,98]; it also plays an interesting role in reprogramming host TCA cycle substrate utilisation [99].

### 2.3. Migration to the Lower Respiratory Tract

These events are intrinsically linked to migration to the LRT, which can mark the next phase in pneumococcal infection. This is not a passive process but one where bacteria actively sense several factors, including temperature (LRT is closer to core body temp than URT) and oxygen partial pressure, as discussed above. A further consideration is nutrient availability. Throughout its colonisation phase, pneumococcus uses glycosidases such as NanA to cleave various sugars from glycoproteins on epithelial surfaces, providing the bacteria with a carbon source [100]. However, in the LRT, the apical surface of these cells is coated in a surfactant that is rich in dipalmitoylphosphatidylcholine (DPPC), a dominant phospholipid that acts as a scaffold for alveolar structure during breathing and reduces surface tension [101,102]. Surfactant proteins A to -D (SP-A-D) are also present to reduce surface tension at the air–liquid interface (B and C) and to bind pathogens for clearance and modulate immune effector function (A and D) [102,103,104]. Pneumococcus expresses a variety of glycosidases (e.g., NanA, BgaA) to strip sugar moieties from the host, which could be used to induce structural deformation and loss of neutralising function in SP-A and SP-D [105,106,107,108].

These modifications to pulmonary surfactant provide a basis for initial epithelial anchoring via CbpA (PspC), allowing efficient colonisation [52,109]. Concurrently, the bacterium begins to release sublytic amounts of pneumolysin (PLY), which was traditionally thought to be released primarily during LytA-triggered cell lysis [110,111]. However, recent reports suggest that the toxin is expressed, exported through the cytoplasmic membrane and “stored” around the PG layer [112,113]. Choline binding proteins (such as CbpA) then distort the PG layer as they interact with the alveolar environment, which releases small doses of PLY in a localised manner, working to dissolve the surfactant layer and provide access to the epithelia. Once established on the apical surface, pneumococcus begins to use the liberated nutrients to rapidly proliferate until a critical population threshold is reached. This is detected by the ComCDE system via accumulation of Competence-Sensing Peptide (CSP) at a specified level [114,115], providing the switch for LytA expression along with the rest of the competence regulon. LytA is the core amidase that controls the autolysis of pneumococcal cells [116], triggering rapid and dramatic PG remodelling that results in cell lysis. This is thought to occur only in a subset of the bacterial population, triggering the release of large volumes of PLY to damage local host epithelia and aid invasion.

PLY release has two functions: (1) to break down the surrounding surfactant layer to allow further colonisation and (2) to stimulate the immune system within the LRT. In humans, this results in high levels of inflammation through the inflammasome (NLRP3) [117,118], whilst the complement cascade results in high levels of C3a and C5a. These act as anaphylatoxins, increasing permeability of the local host cells and causing fluids to leak directly into the alveolar space [119,120]. C5a also recruits neutrophils to the site of infection. Because neutrophils normally reside in the capillaries surrounding the lungs, induction of fluid leakage into the lungs is required for efficient recruitment [121,122]. This is aided by several other chemoattractants that are released via TLR2 (IL-8) [123,124], HXA3 forming a cross-junction gradient [125,126] and formyl peptides released from pneumococcus itself [127,128]. Once recruited, neutrophils undergo a degranulation process upon detection of pneumococcal cell wall components in murine models [129,130,131], releasing components such as elastases and matrix metalloproteases [131]. Together, these activate neighbouring goblet cells to hyper-secrete mucins, forming the basis for sputum and trapping bacteria [132,133].

### 2.4. Trans-Epithelial Migration and Bloodstream Infections

Due to the thick capsule layer and high levels of PLY surrounding the site of infection, pneumococcus often persists in the alveoli, allowing it to move to the next phase of its invasive infection. Through two distinct mechanisms, pneumococcus is able to cross the epithelial barrier, migrating into the blood. First, this can be achieved via a transcellular route, whereby PspC binds the polymeric Ig receptor in a manner that mimics host IgA [134,135] or via clathrin-mediated endocytosis as a result of cell wall-bound ChoP interacting with host platelet-activating receptors [136,137]. Both pathways result in pneumococcus shedding much of its thick capsule and being internalised into epithelial cells, where it is transported to the basal side by host machinery [138,139]. This shedding is thought to be facilitated by LytA remodelling of the cell wall [140]. It should be noted that most pneumococcal cells are destroyed at this stage, but a subset achieve successful migration by exploiting host non-acidified endosomes [139,141]. Once at the basal side of the host cell, PspC interacts with the laminin receptor [136,142] and uses surface proteases such as CbpG and PrtA to digest structural collagen and laminin [143,144]. Second, the paracellular route involves pneumococcus combining the effects of PLY and a secreted hyaluronate lyase together with downregulation of Claudin-7/10 via the TGT-β pathway to reduce the structural integrity of tight junctions [145,146]. This allows the bacterium to get between cells and bypass internal host defences.

Should pneumococcus migrate into the blood, it circulates entirely as an opaque variant (heavily encapsulated), which protects it from opsonisation mechanisms [147]. Once in the bloodstream, pneumococcus is also able to continue scavenging iron through the mechanisms discussed above whilst keeping peroxide levels low [148]. Whilst these mechanisms cause localised damage to host blood cells, they also induce an increase in growth rate, as discussed above. This damage in turn leads to a significant upregulation of TLR2/4 signalling via release of cell wall components, which in turn induces the release of pro-inflammatory cytokines and nitric oxide [149,150]. This triggers systemic vascular fluid leakage from blood vessels as well as smooth muscle relaxation and thus vasodilation. Taken together, these cause blood pressure to drop severely, which may contribute to the development of septic shock.

Furthermore, pneumococcus uses the circulatory system to travel to other areas of the body, using the ChoP and PspC-mediated attachment mechanism described above to colonise other organs. This is of particular concern at the blood–brain barrier, a structure that usually protects the brain from pathogens whilst remaining permeable to blood and small molecules. However, pneumococcus can utilise the same transcellular migration mechanics described above to translocate across this structure to access both the brain and cerebrospinal fluid [141,151]. Once inside the subarachnoid space, the bacterium is able to grow and divide incredibly quickly due to the scarcity of immune defences (antibodies or neutrophils), as well as the nutrient-rich environment [152,153]. As a result, one of the key immune effectors in this niche is the complement cascade, which quickly becomes exponential, leading to severe intracranial inflammation and eventual meningitis [136,154].

## 3. Dynamics of Pneumococcal Co-Infection

Pneumococcal disease has been associated with the circulation of a variety of other pathogens, both bacterial and viral [155,156,157,158]. Henceforth, any relationship that describes two or more pathogens invading a host simultaneously or cocirculating together will be defined as “co-infection”. In general, these co-infections have been shown to trigger more severe disease through synergistic mechanisms that are beginning to be understood. These range from providing the triggers discussed above for pneumococcus to switch from commensal to pathogenic lifestyles to creating a more favourable environment for the bacteria to grow.

### 3.1. Viral Co-Infections

The importance of the relationship between viruses and bacteria in causing disease has been recognised for over a century: bacterial complications were found to be a major cause of death in the 1918 influenza pandemic [159], with pneumococcus found to be the predominant bacterium [160]. Excess cases of IPD have also been shown to correlate with viral circulation in humans [161,162,163,164]. Two broad mechanisms are thought to account for this synergy. First, the virus may render the mucosa more susceptible to new acquisition or increase the density of existing carriage, thereby raising the probability of a stochastic invasive breakthrough. Second, the virus can tip the balance from carriage to invasion itself, by weakening host defences or by enhancing pneumococcal virulence. One possible proposed mechanism for this switch is the changes in temperature influencing the CiaRH system, as described above [75,76]; though this is currently speculative as other factors likely influence responses.

#### 3.1.1. Influenza A Increases Pneumococcal Replication

Influenza is an enveloped, single-stranded, negative-sense RNA virus split into four types, A–D, though influenza D is primarily a cattle virus and will not be discussed here. Of the roughly 1 billion influenza cases worldwide each year, influenza A dominates with around 75% of cases reported, whilst influenza B makes up around 20–25% of cases, with influenza C being extremely rare [165,166]. The virion’s surface features two main proteins for infection and replication: haemagglutinin (HA) and neuraminidase (NA). HA specifically interacts with sialic acid on the surface of host cells, inducing receptor-mediated endocytosis via clathrin lattices [167,168]. NA, by contrast, is responsible for the final stage of viral budding as well as other housekeeping roles. The protein’s main role is to cleave sialic acids post-budding to allow the new virions to escape the host cell, but it also serves to degrade the mucus layer surrounding epithelial cells to allow the virion to bind [169].

In this way, the initial manner of colonisation is shared between pneumococcus and influenza; however, several reports using mouse and human patients have demonstrated that pneumococcal density increases with influenza co-infection [170,171]. Following influenza A challenge, ferrets demonstrate increased susceptibility to pneumococcal acquisition on exposure [172], and ferrets and mice with existing pneumococcal colonisation demonstrated increases in colonisation densities following viral challenge [172,173,174]. This is reflected in human populations in both the UK [175] and The Gambia [176], where pneumococcal colonisation density rose in children following receipt of an intranasal live-attenuated influenza vaccine. In a longitudinal study in Peru, antecedent influenza or parainfluenza infection was found to increase the probability of new pneumococcal detection [177].

Influenza can promote pneumococcal binding when neuraminidase (NA) cleaves terminal sialic acids from host glycans, exposing underlying receptors for bacterial adhesion [178,179]. Inhibition with the NA inhibitor oseltamivir improved survival from secondary pneumococcal infection in mice, independent of the viral load or pathogenicity of the influenza infection [179]. The magnitude scales with NA activity and is strain-dependent, with NA activity correlating both with pneumococcal adherence to human respiratory cells and with mortality in co-infected mice [178]. Beyond receptor unmasking, viral NA activates host TGF-β signalling to upregulate fibronectin and further adhesion receptors [180], while the liberated sialic acid doubles as a carbon source supporting pneumococcal growth and rising colonisation density [181].

#### 3.1.2. RSV Makes Direct Protein–Protein Interactions with Pneumococcus

Respiratory syncytial virus (RSV) is another single-stranded, negative-sense RNA virus that is commonly associated with pneumococcal infections. Human RSV is split into two subtypes; RSV-A is generally seen as historically more severe than RSV-B (higher case fatality rate), which correlates with the latter being slightly more dominant in human outbreaks [182,183]. During infection, the major attachment protein, RSV G, binds to CX3CR1 and heparan sulfate on epithelial cell surfaces [184,185], bringing the fusion protein, RSV F, close to the membrane to facilitate genomic deposition [186]. From here, the RSV N, P and L proteins promote replication via autophagy-mediated inhibition of apoptosis [187,188], which in turn leads to the display of new RSV F on the surface of host cells. In this way, the virus triggers formation of syncytia, large, multinucleated fusions of host cells that ultimately detach from the main epithelium and contribute to pneumonia symptoms [189].

Concurrently, pneumococcus is able to make a direct protein–protein interaction with the major attachment protein, RSV G, using its cell wall synthase, PBP1a [190,191]. Through these proteins, pneumococcus is able to significantly increase its adherence to host cells, leading to higher levels of bacteraemia [190,191]. This interaction also triggers changes in gene expression, specifically upregulation of sugar synthesis for thick capsule production and PLY, both being factors in the transition of pneumococcus to an invasive pathogen [191]. Interestingly, a recent study in Laos suggested that pneumococcal/influenza co-infections led to increased pneumococcal density, whilst a pneumococcal/RSV co-infection did not [192]. This highlights the inherent complexity of these interkingdom interactions and their effects on disease progression.

There is notable epidemiological heterogeneity between different viral–pneumococcal relationships. RSV causes its most severe disease at the extremes of age and in the immunosuppressed [193], and its interaction with pneumococcus generally follows the same age distribution. In young children, RSV and rhinovirus were found to raise the density of pneumococcal carriage, although the authors note that this only accounted for a relatively small proportion of the variation [194]. Pneumococcal carriage dynamics have been shown to vary over the course of paediatric RSV bronchiolitis [195,196]. These carriage effects are mirrored by clinical disease patterns, where RSV’s association with invasive pneumococcal disease is likewise greatest in infants and young children [162,164]. RSV co-infection may also change which pneumococci cause invasive disease. French national surveillance showed that RSV circulation shifted the serotype distribution of paediatric IPD toward serotypes of otherwise low invasive potential; the lower a serotype’s intrinsic disease potential, the more RSV circulation increased its involvement in IPD (Rho = 0.60), suggesting that RSV can enable normally low-virulence serotypes to invade [197].

#### 3.1.3. Viral Promotion of Pneumococcal Immune Evasion

Following mucosal adherence, pneumococcus must evade immune responses to persist and, in some cases, cause invasive disease. The mucociliary escalator is the first barrier pneumococcus encounters, and it can be impaired by respiratory viral infection. In murine trachea, prior influenza reduced tracheal mucociliary velocity and impaired clearance of pneumococci by two hours after challenge [198]. Similar ciliary dysfunction occurs with other respiratory viruses. RSV caused ciliary dyskinesia in human airway epithelial cultures; beat frequency remained intact, while cilia were progressively lost and ciliated cells were damaged [199]. Impaired ciliary function has also been reported with rhinovirus and seasonal and pandemic coronaviruses [200,201]. Additionally, influenza can also compromise barrier integrity by disrupting epithelial tight junctions and increasing permeability [202]. Pneumococci bind extracellular matrix and host adhesive proteins, including laminin, type IV collagen and vitronectin, in vitro [201]. Although viral epithelial damage could theoretically increase access to these ligands, evidence that basement membrane exposure enhances pneumococcal attachment is lacking. Early microscopy supported attachment to denuded murine tracheal surfaces after influenza [203], whereas subsequent murine perfused tracheal experiments found no increased adherence despite extensive denudation, favouring impaired clearance as an explanation for increased bacterial burden [198].

Respiratory viruses can also reshape the innate immune response, blunting both the recruitment and the function of key effector cells. In murine models, antecedent influenza suppresses the early neutrophil influx to the site of pneumococcal challenge, driven largely by type I interferon-mediated downregulation of the neutrophil-recruiting chemokines MIP-2 and KC [204] and increased levels of the anti-inflammatory and immunoregulatory cytokine IL-10 [205]. This recruitment defect is time-dependent, and although later neutrophil accumulation is often preserved or even exaggerated, the neutrophils that do arrive are often functionally impaired, with reduced phagocytosis, respiratory burst and killing [206,207]. RSV produces a comparable outcome, with infected mice showing altered neutrophil function and clearing bacteria less efficiently from the lung [208].

Macrophage responses are compromised by both RSV and influenza. Following influenza, the ability of alveolar macrophages to phagocytose extracellular pneumococci is diminished, driven by interferon gamma-mediated suppression of antibacterial function [209]. RSV likewise diminishes pneumococcal phagocytosis by human macrophages [210] and the bactericidal activity of monocytes [211] in vitro. RSV infection has also been shown to drive Gas6/Axl-mediated macrophage polarisation that exacerbates secondary pneumococcal pneumonia [212]. Together with a dampened local cytokine and chemokine response, these changes leave the airway susceptible to pneumococcal outgrowth and progression to invasive disease. Notably, these mechanisms are not confined to animal models: in an experimental human challenge, influenza-induced inflammation impaired innate immune control of pneumococcal colonisation [213].

#### 3.1.4. Other Respiratory Viral Infections

The existing evidence base for viral–pneumococcal interactions is heavily weighted toward influenza and RSV, although this may reflect research priorities as much as biology. One common mechanism of a synergistic relationship for some viruses is host receptor upregulation. In human epithelial cell cultures, infection with human parainfluenza 3 (HPIV-3) and RSV both increased pneumococcal cell adhesions as well as expression of host adhesion molecule receptors ICAM-1 and CEACAM1. Notably, influenza also increased adhesion but without a notable increase in receptor expression. RSV also increased the expression of the platelet-activating factor receptor (PAF-r), although neither influenza nor HPIV-3 did [214]. The seasonal coronavirus HCoV-NL63 has also been shown to upregulate PAF-r, leading to increased pneumococcal binding in cell lines, although further experiments showing that blockage of this receptor did not reduce pneumococcal adherence led the authors to doubt the relevance of this effect [215].

Rhinovirus infection has also been shown to increase PAF-r expression and consequent increased adhesion in cultured cell lines [216,217]. This is supported by a human pneumococcal challenge study that found that participants who were positive for either rhinovirus or RSV when challenged had a greater chance of acquisition [218]. Additionally, in a longitudinal study of children under five, symptomatic rhinovirus or RSV infection was associated with a modest rise in pneumococcal density [194]. The potential clinical relevance of rhinovirus–pneumococcus interactions is supported by ecological time-series studies linking rhinovirus circulation with IPD. Their model attributes 6.97% (CI 4.27–10.28%) of IPD cases across all ages to rhinovirus, a larger estimate than either RSV or influenza. The strongest estimated contribution was in adults aged 45 years and older [163]. The evidence in younger children is less consistent: in Finnish children under five, IPD rates were approximately twice as high during periods of high versus low rhinovirus circulation [219]; however, a subsequent analysis of Israeli children under five found no significant contribution of rhinovirus to the pneumococcal disease outcomes examined [220].

Adenovirus has been shown to increase pneumococcal adherence in cell lines. Although the mechanism in one study was not established, the findings were consistent with virus-induced upregulation or alteration of epithelial binding sites [221]. The few studies that have examined the clinical association between adenovirus and invasive pneumococcal disease provide a conflicting picture. Although a positive ecological association between adenovirus circulation and IPD in children has been reported in some studies [222,223], the association has not been found in others [220,224]. In chinchilla models examining adenovirus-associated susceptibility to pneumococcal otitis media, one study found a positive effect [225], whereas a later study did not [226], although different adenovirus types were used in the two experiments.

SARS-CoV-2 does not appear to be a significant population-level precipitant of IPD. In children during the COVID-19 pandemic, despite high SARS-CoV-2 transmission, a significant drop in IPD was noted, even with relatively stable pneumococcal colonisation rates. These reductions and the subsequent rebound correlated closely with disrupted seasonal viral transmission [224,227]. However, altered pneumococcal transmission dynamics from non-pharmaceutical interventions, differential attack rates between age groups and ascertainment bias during the pandemic may confound this observation. More direct evidence provides little support for a strong causal pathway from SARS-CoV-2 to subsequent IPD. In England, linkage of IPD to SARS-CoV-2 between February and June 2020 found only 40 simultaneous IPD cases among 160,886 laboratory-confirmed COVID-19 cases, approximately 0.025%. Whilst this argues against SARS-CoV-2 acting as a frequent population-level trigger of IPD, where co-infection did occur, it was associated with a high case-fatality rate [228].

Experimental data nevertheless show that a causal interaction is biologically plausible. Pneumococcal carriage has been associated with attenuation of the antiviral immune response during SARS-CoV-2 infection [229], while murine models demonstrate increased pneumococcal burden and disease severity when pneumococcal challenge followed SARS-CoV-2 infection [230,231]. These findings therefore support the possibility that SARS-CoV-2 can facilitate pneumococcal disease in individual hosts but do not establish that this pathway makes an important contribution to IPD incidence at a population level.

### 3.2. Bacterial Co-Infections

Besides viral interactions, pneumococcus is known to influence and be influenced by a number of bacteria, both pathogens and commensals. Within the initial site of colonisation by pneumococcus, in the URT, the existing microflora create a changeable and highly defence-orientated environment that protects the host from external threats [232]. However, pneumococcus has several mechanisms for outcompeting these bacteria in a local niche (see above), which can be influenced by signals from competing bacteria.

#### 3.2.1. Pneumococcus Directly Alters *Staphylococcus aureus* Behaviour

During the early 2000s, several studies reported a negative association between carriage of pneumococcus and *S. aureus* in human children [233,234]. Studies since then have confirmed this observation, though again mostly in young children [235]. Interestingly, both pneumococcus and *S. aureus* occur as secondary bacterial infections following influenza A infections [236,237]. This negative association may be a form of competition for a niche rather than a direct interaction. Whilst it has been shown that pneumococcal serotype 19F forms a robust biofilm with *S. aureus* UAMS-1 under fixed tissue culture (TC) conditions [238], the authors also demonstrated that UAMS-1 does not disperse from this biofilm in response to heat stimuli. Furthermore, it has been shown that preformed *S. aureus* biofilms can be eradicated within hours of treatment with pneumococcal cultures [239], though the effect is serotype-specific, with clinically relevant serotypes, such as serotype 2, failing to show staphylococcal-specific bactericidal activity. This activity had previously been linked to pneumococcal peroxide production [240], though recent advances point to a direct interplay between the two, with hydrogen peroxide being further converted by pneumococcus to generate toxic hydroxyl radicals [239,241].

#### 3.2.2. Passive Protection of Pneumococcus Through Co-Biofilms

*S. pneumoniae* is known to co-infect with a variety of pathogens, including non-typeable *Hemophilus influenzae* (NTHi) [156,242,243] and *Moraxella catarrhalis*, another commensal that can act as an opportunistic pathogen [244,245]. These cases contrast each other in mechanism of interaction, with NTHi acting more antagonistically against pneumococcus under certain conditions. Both species have been shown to compete during both planktonic growth and active infection in vitro and in humans [156,243]. This appears to be mediated by both strains of bacteria as well as local environmental triggers such as nutrient availability [242,243,246,247,248]. Type IV pili appear to be induced on NTHi only in biofilm conditions, leading to changes in gene expression in pneumococcus that are dependent on this physical contact [246]. The major autolysin LytA, as well as the major virulence factor PLY, appears to be downregulated in this case [249], prompting pneumococcus to remain in a biofilm state. This is associated with changes to the NTHi outer membrane lipid modifications, driven by sialic acid and ChoP additions that help maintain the co-biofilm and protect against complement cascades [250,251,252]. This protection is enhanced by expression of SapA by NTHi, which removes secreted antimicrobial peptides as well as high levels of β-lactamases from the local environment [253,254], helping protect both species from clinical intervention [255,256].

By contrast, pneumococcus has a more cooperative relationship with *M. catarrhalis* (the causative agent of otitis media in humans), compared to the competitive situation with NTHi. Co-biofilms are frequently observed both in vitro and clinically, which is thought to be due to indirect physical attachment whereby pneumococcal PspC/PavA interact with the same host ligands as *M. catarrhalis* UspA1/2 [257,258,259,260] but do not interact with each other. This creates an interwoven biofilm that is maintained by *M. catarrhalis’* degradation of pneumococci’s peroxide using the catalase KatA [261,262]. Furthermore, each bacterium provides passive protection against multiple classes of antibiotics, increasing the difficulty in treating otitis media [244,263,264]. The core Lipid A of *M. catarrhalis* is modified by the actions of LpxXL to reduce permeability to hydrophobic or cationic drugs [265,266] whilst also producing BRO-1/2 β-lactamases to degrade antibiotics [267,268]. These enzymes are not confined to the cell surface but are packaged into outer membrane vesicles (OMVs), allowing protection to be extended across the niche [256]. As a result, co-infection between pneumococcus, NTHi and *M. catarrhalis* is common, often resulting in more serious infection thanks to the protective cooperation between these species [256,262].

#### 3.2.3. Pneumococcal Genetic Hyperplasticity

Another consideration for pneumococcal infection dynamics and interactions with other organisms is the bacterium’s genomic hyperplasticity. *S. pneumoniae* are highly naturally competent, due to the ComCDE operon [114,115]. As discussed above, this system is induced during biofilm establishment as a result of CSP buildup in dense cell populations [23]. This can also occur in situations where the biofilm is mixed-species, in which the system promotes expression of PspC and lysozyme-like proteins LytB and LytC [269,270,271]. These enzymes cleave the cell wall glycans of neighbouring cells, providing pneumococcus with a colonisation advantage against other species. This is aided by the ComM protein, which shields *S. pneumoniae* from this hydrolytic activity by reorganisation of the cell wall, particularly at the septa [272,273].

Concurrently, the act of inducing lysis in neighbouring cells generates the release of DNA from several species, not only pathogens but the commensal microbiota as well. Being naturally competent, pneumococcus can take advantage of this, using the ComGC pilus to bind and internalise DNA [274,275,276]. From here, the SsbB/DprA/RecA complex binds internalised DNA and carries out unbiased homologous recombination events in multiple places along the chromosome [277,278]. In this way, pneumococcus can generate mutations that can require alterations to clinical interventions. This is commonly achieved through construction of mosaic genes, which have not been fully replaced, but in which exogenous DNA has been inserted, thus forming chimeric alleles [279,280]. These can range from adherence machinery such as PspA [281] to cell wall synthases (e.g., PBPs) [282,283,284], as well as capsule genes that generate new serotypes [285]. Many of these polymorphisms are often credited with the generation of MDR and XDR strains [286]. Contributing to this situation, pneumococcus has an intrinsically higher spontaneous mutation rate than other bacteria, which can contribute to the appearance of mutant alleles [287,288].

The prevalence of MDR and XDR pneumococcal strains has increased substantially in recent years [40,41,284,289], making the limited development of new therapeutic options increasingly concerning [290]. As discussed above, vaccination remains the most important intervention against pneumococcal disease, despite the increasing contribution of non-vaccine serotypes to invasive disease [290,291,292,293]. However, both vaccination and antibiotic use exert selective pressures that can reshape the pneumococcal population [294], emphasising the need to continually reevaluate the effectiveness of prevention and treatment strategies. These decisions must also account for co-infection, which can alter the clinical significance of pneumococcal detection and influence appropriate antimicrobial therapy.

## 4. Discussion

Bacterial–viral co-infections and the synergy between them represent a long-established clinical challenge, but determining the relative contribution of viral and bacterial pathogens to an individual patient’s illness remains difficult. This distinction matters because the appropriate use of antibiotics depends heavily on the likelihood that bacteria contribute to the disease process. For example, much of the mortality during the 1918 influenza pandemic is now thought to have resulted from secondary bacterial pneumonia, meaning that effective antibacterial treatment would likely have prevented many deaths. By contrast, bacterial co-infection appears to have been relatively uncommon among patients admitted with COVID-19, with early studies estimating rates of only 2–8% [295,296]. Nevertheless, antibiotics were frequently prescribed, particularly early in the pandemic when the natural history of COVID-19 and the risk of secondary bacterial infection were still uncertain. Subsequent studies found little evidence of benefit from routine empirical antibiotic treatment [297].

These contrasting examples illustrate the difficulty of making treatment decisions when the contribution of bacterial infection is uncertain. Clinical, radiological and laboratory findings can help distinguish bacterial from viral disease, but none is definitive, particularly early in an infection. Therefore, the requirement for a better understanding of bacterial–viral synergy and how that impacts patient care would likely benefit future treatment options. The current knowledge gap is understanding how viral infection changes the behaviour of bacterial pathogens. The traditional model, in which viral infection damages epithelial barriers or impairs host defences and thereby creates an opportunity for secondary bacterial infection, remains important. However, bacterial–viral interactions can also be more direct, with viruses altering bacterial colonisation, metabolism or virulence through physical and molecular interactions. Pneumococcus provides a well-studied example, frequently cocirculating and co-infecting with respiratory viruses including influenza A virus and respiratory syncytial virus (RSV). Both viruses have been reported to interact directly with the pneumococcal surface, through cell wall-associated ligands [298] or protein–protein interactions [191]. How these interactions subsequently alter pneumococcal physiology and virulence, however, remains incompletely understood.

**Figure 2:**
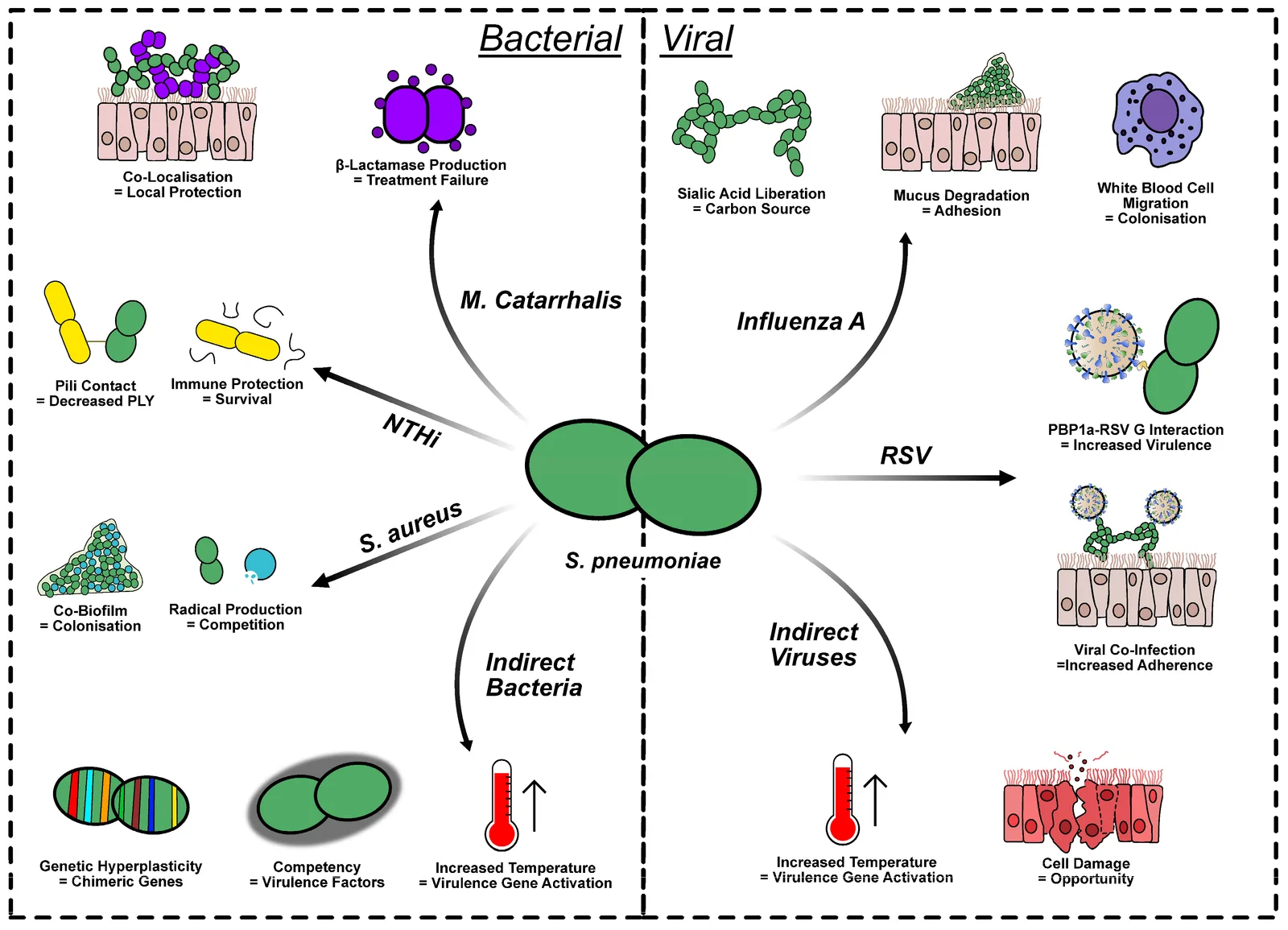
Summary of environmental triggers that control the pneumococcal pathogenicity switch. Labelled arrows denote the likely causative agents discussed throughout this review, with graphical illustrations of the downstream effect(s) on pneumococcus. Each illustration is discussed at length in Section 1, Section 2 and Section 3.

As discussed above, pneumococcus colonises the human URT from a very early age. Several factors are responsible for this, many of which involve a primary viral infection, as summarised in Figure 2. One factor common to both bacterial and viral co-infections could be an increase in temperature, which in turn activates the CiaRH system to trigger downstream virulence responses. This, combined with the increased abundance of sugar molecules liberated through NA activity, providing extra carbon source(s) for pneumococcal growth, allows *S. pneumoniae* to disperse its biofilms and migrate to the LRT. These changes can be exacerbated by direct protein–protein interactions between pneumococcus and virions such as RSV, which have been shown to alter virulence of the bacterium as well as promote bacterial adhesion to the host [191,214]. However, pneumococcal virulence is often characterised as an evolutionary dead end, demonstrating the complexity associated with evolution of interactions between pathogens [52].

While viral co-infection often induces changes in the infection niche, bacterial co-infections appear to present more nuanced mechanisms. *S. pneumoniae* makes extensive use of its genomic plasticity in this sense, exploiting the Com system to both eliminate the competition for nutrients in the colonisation niche and uptake exogenous DNA. This DNA in turn can be integrated into the pneumococcal genome, allowing the bacterium to constantly and rapidly evolve and survive in a challenging environment, developing new resistances and increasing the difficulty of treatment upon infection. Pneumococcus can show antagonistic behaviour when paired with another human opportunistic pathogen such as *S. aureus*. Toxic hydroxyl radicals produced by pneumococcus escape the staphylococcal oxidative stress response and induce bactericidal activity [239,241,299].

Other species, such as NTHi, act as pneumococcal antagonists in planktonic growth [156,243], while a more cooperative interplay is present under biofilm conditions [249]. Pneumococcus, in this case, downregulates both LytA and PLY to remain in a biofilm state and benefit from the protection against antimicrobials and antibiotics given by NTHi expression of SapA and β-lactamases [253,254,256]. Interaction with other bacterial species represents a useful strategy for pneumococcus to avoid clearance and resist treatment whilst improving survival chances, whether by acquiring new resistance or working synergistically with bacterial species that can subvert the activity of certain antimicrobials. These metabolic changes now drive a secondary consideration in terms of treatment. It is well known that the use of broad-spectrum antibiotics leads to increased incidence of AMR globally, as discussed above. However, these treatments also drastically change the selection pressure and landscape of a healthy microbiota, influencing many commensals and other pathogens. Whilst this is most commonly observed in the gut microbiome [300,301], the same principle applies to the URT, favouring certain species of bacteria over others, especially those with inherent β-lactamase expression such as those discussed above. This in turn provides pneumococcus exogenous DNA with which to form new chimaeras and/or biofilm partners.

Overall, the mechanisms described in this review outline the infectious pathway *S. pneumoniae* takes from commensal to virulent pathogen and how these are altered by co-infection to promote pneumococcal virulence. Deepening our understanding of these mechanisms will be critical to the development of novel next-generation treatment options against this global threat.

## 5. Conclusions and Future Perspectives

*Streptococcus pneumoniae* remains a global health threat in vulnerable populations. The switch from nasopharyngeal commensal to invasive pathogen has been described as a highly coordinated, multi-stage process involving environmental triggers that drive changes in molecular mechanisms. This review brings together academic and clinical data to describe the current understanding of pneumococcal infection, as well as the role of co-infection in influencing this process. Many of the mechanisms have been described, providing key insights into the responses of pneumococcus to its environment. However, many interactions are not well defined or are generalised to systems beyond the initial study focus.

One major consideration is the technical bottlenecks that hinder the field. In situ tracing techniques for co-infection are often highly invasive whilst simultaneously lacking the spatial resolution to efficiently track nanoscale co-infection dynamics in real time. As a result, the field relies heavily on end-point analysis to provide a snapshot of the dynamics, which greatly limits understanding. An obvious solution to this is to utilise in vitro culture models with defined input species to ask specific questions of a controlled system. However, these are often limited by the lack of complex 3D architectures that govern many interactions that the pathogen experiences in a live host. Current advances in multi-cell line air–liquid interface models are closing the gap between in vitro and in vivo settings but remain a bottleneck for understanding in their current form. Similarly, animal models have inherent issues, including differences in mucus profiles and receptor affinities for certain pathogens, meaning conclusions from these models may not represent the human host fully.

For pneumococcus specifically, the vast majority of the field has focused on a subset of serotypes (R6, D39, TIGR4), which represents another challenge. Since these strains are covered by existing pneumococcal vaccine programmes, they likely represent a lower proportion of circulating bacteria that are involved in co-infection pathogenesis. It has been noted that different serotypes interact with viruses differently and have different phase variation phenotypes associated with their capsules. It should therefore be noted that some conclusions may not be generalised to all pneumococcal serotypes but are dominated by the behaviour of the most studied strains. Similarly, most studies involving human patients focus on healthy individuals, with the most at-risk populations (children, elderly and immunocompromised) being underrepresented. This challenge makes age-specific factors difficult to discern, including changes in the commensal microbiota composition and immune system, which likely influence host–pathogen interactions.

## Data Availability

No new data were created or analyzed in this study. Data sharing is not applicable to this article.

## Abbreviations

The following abbreviations are used in this manuscript:

WHO	World Health Organisation
IPD	Invasive Pneumococcal Disease
CAP	Community-Acquired Pneumonia
PPV23	Pneumococcal Polysaccharide Vaccine
MDR	Multi-Drug Resistant
XDR	Extensively Drug Resistant
URT	Upper Respiratory Tract
NETs	Neutrophil Extracellular Traps
TM	Transmembrane
PG	Peptidoglycan
RNA	Ribonucleic Acid
UTR	Untranslated Region
RBS	Ribosome Binding Site
PAFR	Platelet-Activating Factor Receptor
ATP	Adenosine Triphosphate
LRT	Lower Respiratory Tract
DPPC	Dipalmitoylphosphatidylcholine
PLY	Pneumolysin
CSP	Competence-Sensing Peptide
DNA	Deoxyribonucleic Acid
ChoP	Phosphorylcholine
TGT-β	Transforming Growth Factor-Beta
HA	Hemagglutinin
NA	Neuraminidase
RSV	Respiratory Syncytial Virus
KC	Keratinocyte-Derived Chemokine
TC	Tissue Culture
NTHi	Non-Typable *Haemophilus influenzae*
OMVs	Outer Membrane Vesicles
PBPs	Penicillin Binding Proteins
AMR	Antimicrobial Resistance
